# Neighborhood deprivation, vehicle ownership, and potential spatial access to a variety of fruits and vegetables in a large rural area in Texas

**DOI:** 10.1186/1476-072X-9-26

**Published:** 2010-05-25

**Authors:** Joseph R Sharkey, Scott Horel, Wesley R Dean

**Affiliations:** 1 Program for Research in Nutrition and Health Disparities, School of Rural Public Health, Texas A&M Health Science Center, MS 1266, College Station, TX 77843-1266 USA; 2 Center for Community Health Development, School of Rural Public Health, Texas A&M Health Science Center, MS 1266, College Station, TX 77843-1266 USA; 3 Program on GIS and Spatial Statistics, School of Rural Public Health, Texas A&M Health Science Center, MS 1266, College Station, TX 77843-1266 USA

## Abstract

**Objective:**

There has been limited study of all types of food stores, such as traditional (supercenters, supermarkets, and grocery stores), convenience stores, and non-traditional (dollar stores, mass merchandisers, and pharmacies) as potential opportunities for purchase of fresh and processed (canned and frozen) fruits and vegetables, especially in small-town or rural areas.

**Methods:**

Data from the Brazos Valley Food Environment Project (BVFEP) are combined with 2000 U.S. Census data for 101 Census block groups (CBG) to examine neighborhood access to fruits and vegetables. BVFEP data included identification and geocoding of all food stores (*n *= 185) in six rural counties in Texas, using ground-truthed methods and on-site assessment of the availability and variety of fresh and processed fruits and vegetables in all food stores. Access from the population-weighted centroid of each CBG was measured using proximity (minimum network distance) and coverage (number of shopping opportunities) for a good selection of fresh and processed fruits and vegetables. Neighborhood inequalities (deprivation and vehicle ownership) and spatial access for fruits and vegetables were examined using Wilcoxon matched-pairs signed-rank test and multivariate regression models.

**Results:**

The variety of fruits or vegetables was greater at supermarkets compared with grocery stores. Among non-traditional and convenience food stores, the largest variety was found at dollar stores. On average, rural neighborhoods were 9.9 miles to the nearest supermarket, 6.7 miles and 7.4 miles to the nearest food store with a good variety of fresh fruits and vegetables, respectively, and 4.7 miles and 4.5 miles to a good variety of fresh and processed fruits or vegetables. High deprivation or low vehicle ownership neighborhoods had better spatial access to a good variety of fruits and vegetables, both in the distance to the nearest source and in the number of shopping opportunities.

**Conclusion:**

Supermarkets and grocery stores are no longer the only shopping opportunities for fruits or vegetables. The inclusion of data on availability of fresh or processed fruits or vegetables in the measurements provides robust meaning to the concept of potential access in this large rural area.

## Introduction

Adequate consumption of nutritious foods, such as fruits and vegetables, is essential for overall good nutritional health, and the prevention and management of nutrition-related health conditions, such as obesity, diabetes, cardiovascular disease, and some cancers [1-8]. Rural populations face some of the same challenges as urban or suburban counterparts but often at a higher degree of severity [9]. Both rural men and women have higher rates of self-reported obesity than men and women in other areas; and rural minorities face an added burden of health risk behaviors based on rural residence and race- and ethnicity-related health disparities [10]. Results from the 1999-2000 National Health and Nutrition Examination Survey (NHANES) indicate that a large proportion of American children and adults do not meet the recommendations for fruit and vegetable intake [11]. Additional studies found low fruit and vegetable consumption among rural populations, especially among low-income and minority subgroups [12,13]. Although the consumption of fruits and vegetables is recommended, they are often not easily accessible [14-16], especially in small-town and rural areas which also lack transportation infrastructure [17]. Consequently, ecological approaches to behavior change and health recognize that there is a dynamic interaction between the individual and where they live [18-21].

**Figure 1:**
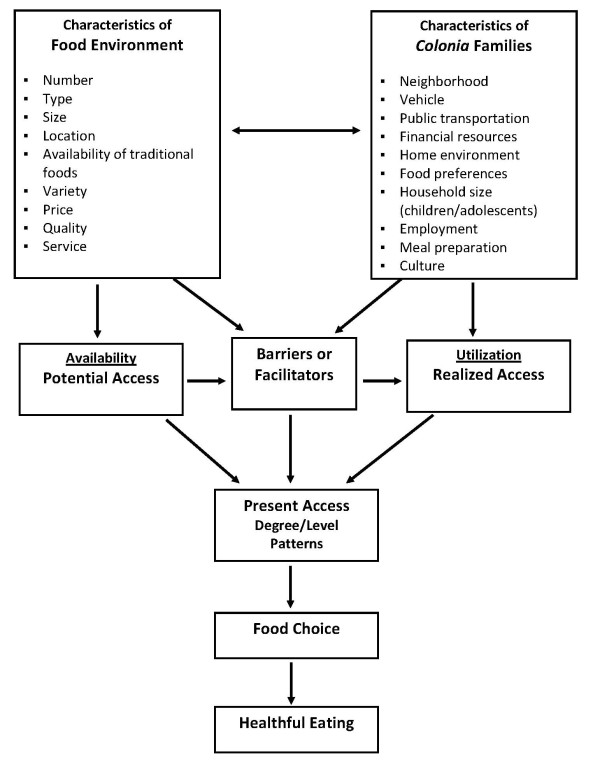
**Conceptual Model of Food Access**.

Various social-ecological frameworks have been used to explain the influence of environment - physical, social, and economic - on individual behaviors [18,19,22,23]. Specifically, social-ecological approaches to food choice and healthful eating recognized that access to food stores may have an effect upon health and well-being, as well as adherence to dietary recommendations [24-27]. The conceptual model in Figure 1, which is based on work in access to healthcare [28,29] provides a framework for understanding food access. This model shows access to healthful food is the result of the relationship between the retail food environment and potential consumers, and suggests food choice and healthful eating are influenced by available (potential access) and utilized (realized access) shopping opportunities. Characteristics of the food environment include: number, type, size, and location of food stores; availability (supply) of food categories (e.g., fresh fruits); and variety of different items within a category (e.g., different types of fresh fruits); price and quality of food items. Characteristics of potential consumers include neighborhood of residence, availability of a vehicle, public transportation, financial resources (type, amount, and timing), home environment (food storage, meal preparation area, and refrigeration), food preferences, meal preparation knowledge and skills, household size, employment, culture, and health. Barriers or facilitators associated with the food environment and/or consumer influence the selection of food purchase opportunity at a given time. For example, limited household refrigeration may require frequent, costly trips for perishable food items; or purchase of more expensive or less healthy food items from a retail store closer to home [14,30,31]. As a result, proximity to food stores may influence food choice through food cost and availability [24,25,27,32,33].

Research on geographic or spatial access from the home to food stores describes a more recent and growing approach to understand access to a variety of healthy foods and to eliminate inequalities in nutrition-related health conditions [20,34,35]. There are various measures that have been used to describe different dimensions of accessibility to food stores. The approach most prevalent in the literature is a measure of proximity or distance (straight-line or network) to the nearest food store [17,36-39]. Other dimensions include coverage (number of food stores within a specified distance or buffer area) [27,37], variety (average distance to the three closest different chain-name supermarkets) [37], and density (proportion or ratio of food stores per county, Census tract, or Census block group) [24,25].

U.S. studies have documented better-quality diets for individuals who reside in closer proximity of supermarkets [40,41]; better access and availability of produce was associated with greater intake of fruits and vegetables [24,33,42-44]. However, physical access may be a major problem for people in deprived or rural communities, especially those without cars, the elderly, and people on low incomes [41,45-49]. In contrast to these studies, research in the U.K. found no association between distances to the nearest supermarket and fruit or vegetable consumption [50], or inconsistent results between the introduction of a new supermarket and consumption [51,52].

There is strong evidence that residents of small-town and rural areas are affected by poor access to supermarkets and healthy food items [17,36,53-58]. In the only U.S. study of neighborhood deprivation and proximity to food stores in a rural area, investigators found that more socioeconomically-deprived neighborhoods (defined by Census block group) had relatively better potential access to retail food stores compared with other neighborhoods [36]. The distance to the nearest supermarket was still beyond usual walking distance.

The preponderance of published work on food access focused on supermarkets and occasionally grocery stores [17,37-40,49,56,59-61]. This limited focus ignores changing market factors that extend beyond supermarkets [34]. The "true" availability of healthy foods may be underestimated, since some of these foods may be available in convenience and non-traditional food stores [17,58]. Traditional food stores, such as supermarkets and grocery stores, are facing increased competition from supercenters, convenience stores, and non-traditional food stores [62,63]. Non-traditional formats, such as drug stores, mass merchandisers, and dollar stores, have perfected "channel blurring" with the rapid expansion of food items to their customary non-food format [64,65]. Over the past 10 years, non-traditional food stores have increased the variety of shopping and food options with the introduction of refrigerated and frozen sections to their stores and lower food prices to consumers, all at the expense of traditional supermarkets and grocery stores [63-65]. While the opportunities for lower priced food items, especially for low-income families, have multiplied, these increased opportunities do not necessarily provide improved opportunities for healthier alternatives [58]. Rural areas are most affected by these changing market forces, where distance and transportation become even more of a factor [17,66-68].

In a broader sense, accessibility could be defined as potential access to healthier foods, with availability (food items present and ready for purchase) having a greater influence on food choice and consumption [69]. Little is known about the influence of changing market factors, such as the expansion of food offerings by mass merchandisers and dollar stores, on access to and availability of healthy foods, especially in rural areas. The U.S. Department of Agriculture's food guidance system in its dietary recommendations for fruits and vegetables identify canned, frozen, and 100% juice in addition to fresh as a way to help people achieve the recommended variety and amount of fruits and vegetables [70]. Studies, for the most part, have limited their investigations to the availability of fresh fruits and vegetables, which ignore the nutrient benefits of canned and frozen fruits and vegetables [71-75]. In a comprehensive review of fresh, frozen, and canned fruits and vegetables, Rickman and colleagues reported that freezing and canning processes may preserve nutrient value that may be lost in fresh products during storage and cooking [74]. In a 1997 study, Klein and Kaletz found that canned and frozen fruits and vegetables are at least nutritionally comparable to fresh [69]. They further confirmed that canned foods may be sometimes better than fresh and frozen varieties in their nutritional contribution to the diet. In addition, all canned fruits and fruit juices contribute less than two percent of added sugars in most American's diet and vegetables contribute less than one percent of sodium [76,77].

Nutritional and health disparities faced by low-income families in small-town and rural areas throughout the world make understanding the effects of changing market factors on access to a variety of fruits and vegetables especially critical [78,79]. This study expands our understanding, both within the United States and internationally, of potential spatial access to a variety of fruits and vegetables by small-town and rural residents by 1) describing the availability (supply) of fresh and processed fruits and vegetables in traditional, convenience, and non-traditional food stores; 2) determining network-based potential access to fresh and processed fruits and vegetables using proximity and coverage criteria for access; and 3) examining the relationship of between neighborhood inequalities (e.g., socioeconomic deprivation and vehicle ownership) and potential access to fresh and processed fruits and vegetables. This is important, considering that 14% of Supplemental Nutrition Assistance Program (SNAP) benefits were not redeemed at supermarkets or large grocery stores and were used to purchase less noncanned fruits or vegetables [17]. Further, the inclusion of fresh and processed fruits and vegetables from all traditional, convenience, and non-traditional food stores is applicable for an international audience that is concerned with geographic inequities in access to a variety of healthy foods.

## Methods

### Geographic setting

**Figure 2:**
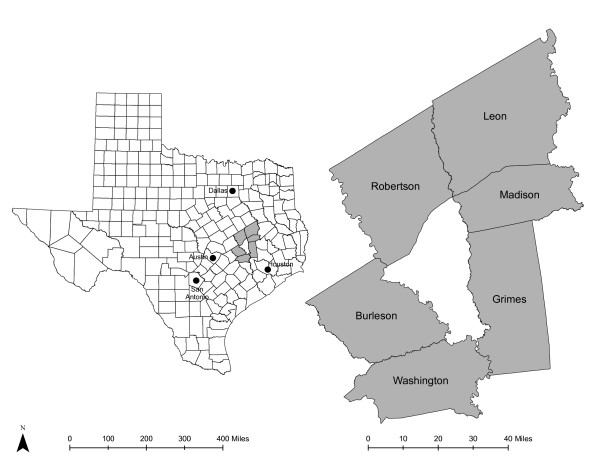
**Map of Texas and Brazos Valley Counties**.

The study used data from the 2006-2007 Brazos Valley Food Environment Project (BVFEP), which was approved by the Institutional Review Board at Texas A&M University, and the decennial 2000 U.S. Census Summary File 3 (SF-3) for six rural counties in the Central Texas Brazos Valley region (see Figure 2). The BVFEP has a history of working with community partners in the seven-county Brazos Valley region of Texas (one urban and six rural counties); all six rural counties were included in this study. These counties, which consist of 101 Census block groups (CBGs) and include five urban clusters (population >2,500), are considered rural based on population density [80,81]. The rural region covered a land area of 4,466 m^2 ^and included a population of 119,654 people [82]. According to the 2000 U.S. Census at the area-level of CBG, the median proportion of minority resident was 24.6% (range 3.6%-89.8%); unemployment was 2.4% (range 0%-8.8%); median of 37.9% of households (range 0%-72.8%) reported an income under 200% federal poverty level (FPL); a median of 14.2% (range 0%-39.8%) completed less than nine years of education; 49.4% (range 0%-71.8%) of housing owner-occupied; median income of $32,269 (range $8235-$51,776), and 37% (range 0%-74.8%) travel at least 30 minutes to work [83]. More than 41% of the 101 CBG are considered low-income areas; that is, areas in which at least 40% of residents has income at or below 200% FPL [17]. Regular public transportation services, such as fixed route, commuter, or taxi services, were not available in the study area [84,85].

### Neighborhood (area-level) inequalities

The CBG, which is the smallest unit of Census geography for which the detailed "long-form" social and economic data from the Census are tabulated, was selected to define a neighborhood [36,86]. ***Neighborhood social and material deprivation***. We applied the Neighborhood Socioeconomic Deprivation Index to each of the 101 CBG in the rural study area [36]. This measure of compound social and material deprivation was calculated from the 2000 U.S. Census Summary File 3 (SF-3) and included unemployment (persons age 16 years and older in the labor force who were unemployed and actively seeking work), poverty (persons with incomes below the federal poverty level), low education attainment (persons age 25 years and older, with less than a 10^th^-grade education), household crowding (occupied households with more than one person per room), public assistance (households receiving public assistance), vehicle availability (occupied housing with no vehicle available), and telephone service (occupied housing with no telephone service). Based on the distribution of scores for the index, a three-category variable for overall neighborhood socioeconomic deprivation was constructed and assigned to each CBG: low deprivation (highest overall socioeconomics and lowest quartile of deprivation scores), middle deprivation (middle two quartiles), and high deprivation (lowest overall socioeconomics and highest quartile of deprivation scores) [36]. ***Vehicle ownership***. Data from SF-3 were used to determine CBG-level vehicle ownership (occupied housing with vehicle available). Tertiles were used to construct a three-category variable for vehicle ownership (range of 62% to 100%); <90.5% of households with a vehicle was considered low ownership, 90.5% to 95.4% medium, and >95.4% high vehicle ownership.

### Food store data

In the 2006 Brazos Valley Health Assessment, random digit dialing methodology was used to recruit adults from the six rural counties in this study for a mailed survey [Wendel, Alaniz, Burdine, Sharkey, Felix, Windwehen: Regional Efforts Aimed at Health Equity: A Case Study in the Brazos Valley, submitted]. More than 1,400 participants responded to a question that asked in what type of store they buy most of their groceries. Almost 80% identified a supermarket or warehouse store; 15.9% a small grocery store; and 4.8% identified a convenience store or other. The BVFEP used ground-truthed methods in a two-stage approach to determine the access to and availability of fruits and vegetables to residents of the 101 CBG. In the first stage, trained observers systematically drove all highways (Interstate, U.S., and State), farm-to-market roads, and city or town streets/roads within the study area. All traditional (supercenters, supermarkets, and grocery stores), convenience (convenience stores and food marts), and non-traditional (dollar stores, mass merchandisers, and pharmacies) food stores were enumerated through direct observation and on-site determination of geographic coordinates using a Bluetooth Wide Area Augmentation System (WAAS)-enabled portable Global Positioning System (GPS) receiver and the World Geodetic System 1984 datum [34,36]. In the second stage, an observational survey instrument was developed, tested, and administered in all food stores by trained observers to determine the availability and variety of fruits and vegetables [58]. Definitions used to classify specific types of food stores are shown in Table 1.

**Table 1:** Definition of types of food stores used in this study

	***Supercenters or superstores***	Very large stores that primarily engage in retailing a general line of groceries in combination with general lines of new merchandise, such as apparel, furniture, and appliances (e.g., Super Wal-Mart, Super Kmart).
	***Supermarkets***	Primarily engage in retailing a general line of food, supermarkets are larger in size (>20,000 sq ft), number of employees, and sales volume [98]. Chain store identification and number of parking spaces (>100) were used to distinguish supermarkets from grocery stores [65,108].
	***Grocery stores***	Primarily engage in retailing a general line of food, grocery stores are smaller in size, not identified as a chain store and have fewer than 100 parking spaces.
	***Convenience stores or food marts***	Primarily engage in retailing a limited line of goods that generally includes milk, bread, soda, and snacks. The convenience store category also included convenience stores with gasoline and gasoline stations with convenience stores.
	***Mass merchandisers***	Large, general merchandise "value" stores, such as Kmart, Target, and Wal-Mart.
	***Dollar stores***	Limited-price general merchandise "value" stores, such as Dollar General or Family Dollar [65,93].
	***Pharmacies and drug stores***	Pharmacies and drug stores that were part of national chains (e.g., CVS, Walgreens).

### Measurement of fruits and vegetable availability

The availability of fruits and vegetables was separately determined from the presence and variety of fresh and processed fruits and vegetables [30,58]. Processed fruits and vegetables included healthier canned, frozen, and juice [74]. Healthier forms of processed fruits included fruits canned in natural juice, fruits canned in light syrup, frozen fruits without added sugar, and 100% fruit juice. Healthier forms of processed vegetables included vegetables canned or frozen without oil or a sauce and 100% vegetable juice. Variety was operationalized as the number of different food items within a fruit or vegetable category (e.g., number of different fresh fruits or number of different types of canned fruits in natural juice).

#### Overall fruit score

Separate scores were constructed to reflect a total of different types of fresh fruits (0 = none, 1 = 1-4, and 2 = ≥ 5); canned fruits in natural juice (0 = none, 1 = 1-4, and 2 = ≥ 5); canned fruits in light syrup (0 = none, 1 = 1-4, and 2 = ≥ 5); frozen fruits (0 = none, 1 = 1-4, and 2 = ≥ 5); and 100% fruit juice (0 = none, 1 = any). A summary score for overall fruits was created by summing the category scores for the number of varieties of fresh fruits, canned fruits in natural juice, canned fruits in light syrup, frozen fruits, and 100% fruit juice. Overall fruit scores range from 0 (worst availability of fruits) to 9 (best availability of fruits). Because the overall fruits score was highly skewed, a three-category variable was constructed for level of overall fruits availability: poor availability (lowest tertile; fruits score 0-1), medium availability (second tertile; fruits score 2-3), and good availability (highest tertile; fruits score 4-9).

#### Overall vegetable score

The overall vegetable availability score combines variety and the presence of a dark green vegetable (e.g., broccoli, collard greens, kale, spinach, or turnip greens) [70]. Separate scores were constructed from a total of different fresh vegetables (0 = none, 1 = 1-4 and no dark green vegetable, 2 = ≥5 and no dark green vegetable or 1-4 and a dark green vegetable, 3 = ≥5 and a dark green vegetable); canned vegetables (0 = none, 1 = 1-4 and no dark green vegetable, 2 = ≥5 and no dark green vegetable or 1-4 and a dark green vegetable, 3 = ≥5 and a dark green vegetable); frozen vegetables (0 = none, 1 = 1-4, and 2 = ≥ 5); and 100% vegetable juice (0 = none, 1 = any). Overall vegetable scores range from 0 (worst availability of vegetables) to 10 (best availability of vegetables). A three-category variable was constructed for level of overall vegetable availability: poor availability (vegetable score: 0-1), medium availability (vegetable score: 2-3), and good availability (vegetable score: 4-10).

### Potential Spatial Access

The population-weighted centroid, which is a more accurate measure than the geographic centroid [36], for each of the 101 CBG was calculated using the ArcGIS Desktop tool Mean Center (Version 9.2, Environmental Systems Research Institute). This tool constructs the CBG mean center based on the mean-weighted x and y values of the block population centroids [36]. Two criteria of potential spatial access were calculated from each CBG [36,87]: 1) proximity, and 2) coverage [37]. Proximity was chosen since it is typically used to measure distance to the nearest food store. Coverage adds the dimension of variety and competition within a specific distance and is not limited to the food stores within an administratively-defined area, such as CBG or Census tract. ***Proximity***: ESRI's Network Analyst extension in ArcInfo 9.2 was used to calculate the shortest network distance along the road network between two sets of paired point data: neighborhood (population-weighted CBG centroid) and the nearest corresponding food store within the six-county study area. The 2003 Tele Atlas Dynamap Transportation version 5.2 provided network data. Separate distances were calculated from each CBG to the nearest supercenter, supermarket, grocery store, convenience store, mass merchandiser, dollar store, and pharmacy in miles. ***Coverage***: Network Analyst computed the total number of each type of food store within one, three, five, and 10 miles, using the shortest network distance from the population-weighted center of each CBG. Since the study area is not a large, highly dense urban area as much of the limited literature describes (e.g., Chicago, Detroit, Montreal, Los Angeles) [32,37,38], coverage distances were selected that represented a long walk (1 mile) and reachable by car (within 3, 5, and 10 miles). Proximity measured the shortest distance needed to travel to a specific type of food store, while coverage indicates the number of opportunities. More opportunities equates to greater accessibility [88]. Proximity and coverage measures were calculated for all traditional, convenience, and non-traditional food stores with a good selection of fresh fruits or vegetables, and food stores with a good selection of fresh or processed fruits or vegetables.

### Statistical analysis

Release 11 of Stata Statistical Software was used for all statistical analyses; *p *< 0.05 was considered statistically significant. Descriptive statistics were estimated for availability of fresh and processed fruits and vegetables by type of food store. Nonparametric tests for trend were estimated across categories of increasing neighborhood deprivation [89]. Distances from the population-weighted centroid of each CBG to the nearest supermarket, traditional food store (supercenter, supermarket, or grocery store), convenience store, and non-traditional (dollar store, mass merchandiser, and pharmacy) food store were calculated. Distance to the nearest supermarket was compared with the distance to the nearest food store with a good variety of fresh fruits, fresh vegetables, fresh and processed fruits, or fresh and processed vegetables by testing for equalities in mean, median, and distribution of distance measures, using Wilcoxon Matched-Pairs signed-rank test. This is a nonparametric test to determine differences between groups of paired data. The null hypothesis is that there is no difference between both distributions. Finally, three multivariate regression models were fitted to determine the relationship of neighborhood deprivation or vehicle ownership to potential spatial access to a good variety of fruits and vegetables, controlling for population density: 1) proximity, 2) 3-mile coverage, and 3) 5-mile coverage. In multivariate regression, several dependent variables are jointly regressed on the same independent variables. The multivariate model approach was chosen instead of four separate multiple regression models (one for each outcome variable) for two reasons: 1) the four outcome variables are correlated with each other and the multivariate regression accounts for this correlation when testing hypotheses about the predictor variables; and 2) the final collection of models is easier to interpret if the same predictor variables are identified.

## Results

### Neighborhood characteristics and food stores

Table 2 shows the distribution of neighborhood socioeconomic characteristics and high levels of neighborhood need in the study area, which are presented as mean and standard deviation for the overall study area and by area-level (CBG) deprivation. There were 26 CBG (25.7%) in the five urban clusters (population range 3,181 to 11,952); 61.5% (*n *= 16) were considered high deprivation neighborhoods; and 81.8% of supermarkets (*n *= 9) were located in five urban clusters [36]. The percent of all socioeconomic characteristics increased significantly with increasing levels of deprivation. Table 2 also shows the distribution of proximity and coverage measures to supermarkets, traditional, convenience, and non-traditional food stores. Access to the nearest food store and the number of the food stores improves with increasing deprivation. Table 3 shows characteristics of CBG that are at least 10 miles one-way from the nearest supermarket or traditional food store. Residents in 47.5% of the 101 neighborhoods (CBG) and 39.3% of rural residents had to travel at least 10 miles; 26.7% of CBG and 20.7% of the population were at least 15 miles from a supermarket; and 12.9% of CBG and 10.5% of population were at least 20 miles from a supermarket. More than 40% of low-income CBG were at least 10 miles from nearest supermarket.

**Table 2:** Neighborhoods characteristics and spatial accessibility to traditional, convenience, and non-traditional food stores by neighborhood socioeconomic deprivation, using measures of proximity and coverage*

	All Deprivation (*n *= 101)	Low Deprivation (*n *= 26)	Medium Deprivation (*n *= 48)	High Deprivation (*n *= 27)
***Socioeconomic characteristics***^†^				
Unemployment,%	2.8 ± 1.9	2.3 ± 1.5	2.7 ± 1.8	3.5 ± 2.1^‡^
Income < 100% FPL,%	16.0 ± 9.6	10.0 ± 4.4	13.3 ± 4.6	26.5 ± 11.5^¶^
Low education,%	15.2 ± 7.2	9.9 ± 3.7	15.0 ± 5.7	20.6 ± 8.2^¶^
Crowded households,%	5.7 ± 5.1	3.2 ± 2.7	4.8 ± 3.8	9.8 ± 6.3^¶^
Public assistance,%	2.9 ± 3.0	1.5 ± 1.8	2.8 ± 2.8	4.7 ± 3.7^¶^
No vehicle available,%	8.9 ± 7.9	3.7 ± 2.7	6.8 ± 3.9	17.5 ± 9.9^¶^
No telephone service,%	4.9 ± 3.9	3.2 ± 2.4	4.4 ± 3.0	7.4 ± 5.1^¶^
***Population density***	353.7 ± 755	153.5 ± 672.6	235.8 ± 613.4	756 ± 918^¶^
**SPATIAL ACCESSIBILITY**				
***Proximity, mi***				
Supermarket	9.9 ± 8.5	11.4 ± 8.8	12.1 ± 8.0	4.7 ± 6.8^‡^
Traditional food store	7.0 ± 6.3	9.4 ± 6.8	8.2 ± 5.8	2.5 ± 4.3^‡^
Convenience store	3.1 ± 2.5	3.7 ± 2.7	3.8 ± 2.3	1.2 ± 2.0^‡^
Non-traditional food store**	8.0 ± 6.5	9.5 ± 6.8	9.2 ± 6.0	4.3 ± 6.0 ^§^
***Coverage - 1 mi***				
Supermarket	0.32 ± 0.58	0.23 ± 0.59	0.12 ± 0.39	0.74 ± 0.66^‡^
Traditional food stores^#^	0.45 ± 0.75	0.27 ± 0.67	0.25 ± 0.70	1.0 ± 0.68^‡^
Convenience stores	1.9 ± 2.9	1.3 ± 2.8	0.79 ± 1.8	4.5 ± 3.0^‡^
Non-traditional food store**	0.55 ± 1.0	0.58 ± 1.4	0.27 ± 0.71	1.0 ± 1.0^§^
***Coverage - 3 mi***				
Supermarket	0.67 ± 1.0	0.58 ± 1.1	0.29 ± 0.74	1.4 ± 1.1^‡^
Traditional food stores^#^	0.89 ± 1.1	0.61 ± 1.1	0.54 ± 0.99	1.8 ± 0.97^‡^
Convenience stores	5.3 ± 7.2	4.7 ± 7.5	2.8 ± 4.9	10.4 ± 7.8^‡^
Non-traditional food store**	1.1 ± 1.7	1.0 ± 1.8	0.52 ± 1.2	2.3 ± 1.8^§^
***Coverage - 5 mi***				
Supermarket	0.83 ± 1.1	0.81 ± 1.2	0.5 ± 0.87	1.4 ± 1.1^‡^
Traditional food stores^#^	1.2 ± 1.1	0.92 ± 1.3	0.94 ± 1.1	1.8 ± 0.97^‡^
Convenience stores	7.3 ± 8.1	7.5 ± 9.4	4.9 ± 6.1	11.2 ± 8.6^§^
Non-traditional food store**	1.4 ± 1.8	1.4 ± 2.1	0.88 ± 1.4	2.3 ± 1.8^¶^
***Coverage - 10 mi***				
Supermarket	1.4 ± 1.1	1.4 ± 1.2	1.1 ± 0.97	1.8 ± 0.91
Traditional food stores^#^	2.1 ± 1.2	2.0 ± 1.4	1.9 ± 1.1	2.5 ± 0.89
Convenience stores	13.8 ± 9.8	15.4 ± 11.2	11.9 ± 8.5	15.5 ± 10.2
Non-traditional food store**	2.2 ± 1.7	2.5 ± 1.9	1.8 ± 1.5	2.6 ± 1.6

FPL = Federal Poverty Level. * Values calculated for each of the CBG (census block group) in the study area (*n *= 101). Proximity determined by the network distance from each CBG population-weighted centroid to the nearest food store; coverage is determined by the number of food stores within a specific network-based distance. Distance (proximity), numbers (coverage), and percentages (socioeconomic characteristics are shown as mean ± standard deviation overall and by category of deprivation.

^†^Items included in the Neighborhood Socioeconomic Deprivation Index

Level of statistical significance for test for trend across ordered groups of neighborhood socioeconomic deprivation: ^‡^*p *< 0.05 ^§^*p *< 0.01 ^¶^*p *< 0.001

^#^Traditional food stores include all supercenters, supermarkets, and grocery stores

**Non-traditional food stores include all dollar stores, mass merchandisers, and pharmacies that sell food items

**Table 3:** Percent and number of CBG at least 10 miles from a supermarket or traditional food store

	≥10 miles		≥15 miles	≥20 miles
	supermarket	Traditional*	supermarket	supermarket
CBG (*n *= 101)	47.5 (48)	32.7 (33)	26.7 (27)	12.9 (13)
% Vehicle Ownership^†^				
Low (*n *= 34 CBG)	29.4 (10)	17.6 (6)	17.6 (6)	5.9 (2)
Medium (*n *= 32 CBG)	59.4 (19)	40.6 (13)	40.6 (13)	18.7 (6)
High (*n *= 35 CBG)	54.3 (19)	40.0 (14)	22.9 (8)	14.3 (5)
Low Income CBG (n = 42)	40.5 (17)	28.6 (12)	19.1 (8)	4.8 (2)
Total Population (119,654)	39.3 (47,039)	27.0 (32,342)	20.7 (24,744)	10.5 (12,519)
Socioeconomic Deprivation				
Low (*n *= 27 CBG)	55.6 (15)	44.4 (12)	29.6 (8)	18.5 (5)
Medium (*n *= 48 CBG)	58.3 (28)	39.6 (19)	31.2 (15)	14.6 (7)
High (*n *= 27 CBG)	18.5 (5)	7.4 (2)	14.8 (4)	3.7 (1)

CBG = census block group * Traditional food store = supercenter, supermarket, or grocery store

^† ^Percent of CBG occupied households with access to a vehicle: Low = <90.5%; Medium = 90.5-95.4%; and High = >95.4%.

### Availability of fruits and vegetables

Observational surveys were completed in 185 (99.5%) food stores; one convenience store refused to participate. Table 4 shows the availability and variety of fresh and processed fruits and vegetables by food store type. As a group, convenience stores provided less availability of fruits and vegetables than traditional or non-traditional food stores. Almost 100% of non-traditional food stores (i.e., dollar stores, mass merchandisers, and pharmacies) offered processed fruits and vegetables. A greater percentage of convenience stores offered processed vegetables rather than processed fruits. The data show that variety of fresh or processed fruits and vegetables was better in supermarkets compared with grocery stores, and in dollar stores compared with convenience stores. Summary scores for availability and variety of fruits and vegetables, which combine fresh and processed fruits or vegetables show that dollar stores offered a greater variety of fruits than either convenience stores or mass merchandisers; variety of vegetables was greater at supermarkets compared with grocery stores. Table 5 shows the level of availability and variety of fruits and vegetables, based on a summary score of the number of different types of fruits or vegetables present. All traditional food stores marketed a good variety fruits and vegetables. More than 43% of dollar stores and 10% of convenience stores offered a good variety of fruits and almost 94% of dollar stores and 28% of convenience stores displayed a good variety of vegetables.

**Table 4:** Scores for availability and variety of fresh and processed fruits and vegetables by food store type (*n *= 185)

	*Traditional Food Stores*			*Convenience Food Stores*	*Non-Traditional Food Stores*		
	Supercenter (*n *= 1) %	Supermarket (*n *= 11) %	Grocery (*n *= 12) %	Convenience (*n *= 140) %	Dollar (*n *= 16) %	Mass (*n *= 4) %	Pharmacy (*n *= 1) %
***Fruits***							
Fresh							
0	0	0	0	85	100	100	100
1 = 1-4	0	0	25.0	14.3	0	0	0
2 = ≥5	100	100	75.0	0.7	0	0	0
Processed							
Canned in natural juice							
0	0	0	0	63.6	25.0	0	0
1 = 1-4	0	0	33.3	33.6	75.0	100	100
2 = ≥5	100	100	66.7	2.9	0	0	0
Canned in light syrup							
0	0	0	0	65.7	6.3	50.0	0
1 = 1-4	0	0	41.7	32.1	25.0	50.0	100
2 = ≥5	100	100	58.3	2.1	68.7	0	0
Frozen							
0	0	9.1	16.7	95.7	100	100	100
1 = 1-4	0	0	58.3	4.3	0	0	0
2 = ≥5	100	90.9	25.0	0	0	0	0
100% Fruit Juice							
0	0	0	0	3.6	0	0	0
1 = ≥1	100	100	100	96.4	100	100	100
***Vegetables***							
Fresh^¶^							
0	0	0	0	85.7	100	100	100
1	0	0	8.3	11.4	0	0	0
2	0	0	58.3	2.9	0	0	0
3	100	100	33.3	0	0	0	0
Processed							
Canned*							
0	0	0	0	25.0	6.2	0	0
1	0	0	0	26.4	0	50.0	100
2	0	0	0	20.0	0	25.0	0
3	100	100	100	28.6	93.8	25.0	0
Frozen							
0	0	0	0	90.0	31.2	75.0	100
1 = 1-4	0	0	0	7.1	68.8	25.0	0
2 = ≥5	100	100	100	2.9	0	0	0
100% Vegetable Juice							
0	0	0	0	24.3	25.0	0	100
1 = ≥1	100	100	100	75.70	75.0	100	0
***Summary Score***^†^							
Fruit	9	8.8 ± 0.6^¶^	7.1 ± 1.7	1.9 ± 1.1	3.4 ± 0.6^‡^	2.5 ± 0.6	3
Vegetables	9	9 ± 0^¶^	8.2 ± 0.6	2.6 ± 1.8	4.2 ± 1.0^‡^	3 ± 1.4	1

^‡^100% juice

*0 = none; 1 = 1-4 vegetables (none are dark green vegetables); 2 = 1-4 vegetables (one dark green) or 5-9 vegetables (none are dark green); 3 = ≥5 vegetables (one dark green).

^†^Summary scores were created separately for fruit and vegetables by summing category scores for fresh and processed fruits or vegetables (reported as mean ± SD).

^‡^Statistically significant from convenience stores (*p *< 0.001) for fruits and vegetables (*p *< 0.01); and statistically significant from mass merchandisers for fruit (*p *< 0.05).

^¶ ^Statistically significant from grocery stores (*p *< 0.001)

**Table 5:** Level of availability and variety of fresh and processed fruits and vegetables by food store type

	*Traditional Food Stores*			*Convenience Food Stores*	*Non-Traditional Food Stores*		
	Supercenter (*n *= 1) %	Supermarket (*n *= 11) %	Grocery (*n *= 12) %	Convenience (*n *= 140) %	Dollar (*n *= 16) %	Mass (*n *= 4) %	Pharmacy (*n *= 1) %
Fruit*							
Poor	0	0	0	50.7	0	0	0
Medium	0	0	0	39.3	56.2	100	100
Good	100	100	100	10	43.8	0	0
***Vegetable***^†^							
Poor	0	0	0	32.1	6.2	0	100
Medium	0	0	0	40.0	0	75.0	0
Good	100	100	100	27.9	93.8	25.0	0

* Fruit availability: Poor = summary score 0-1; medium = summary score 2-3; good = summary score 4-9

^† ^Vegetable availability: Poor = summary score 0-1; medium = summary score 2-3; good = summary score 4-10.

### Potential spatial access to fruits and vegetables

**Figure 3:**
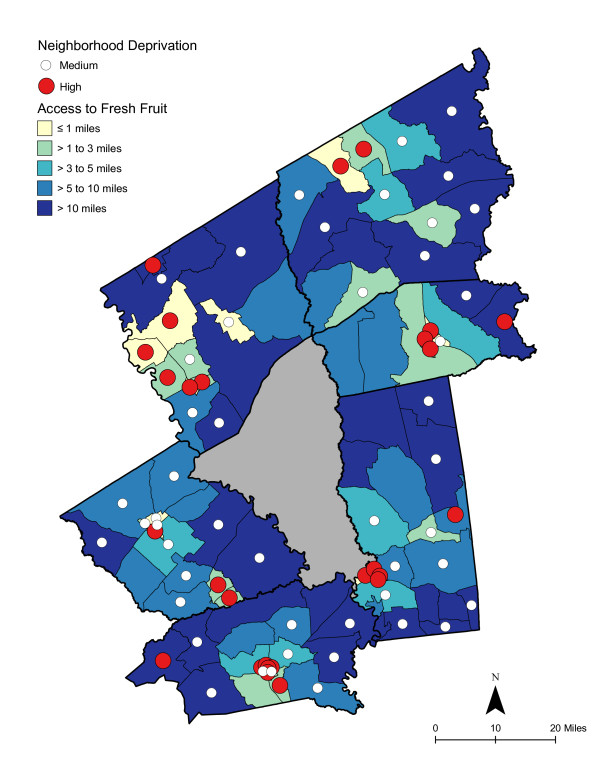
**Area-level (CBG) Deprivation and Access to Fresh Fruit**.

**Figure 4:**
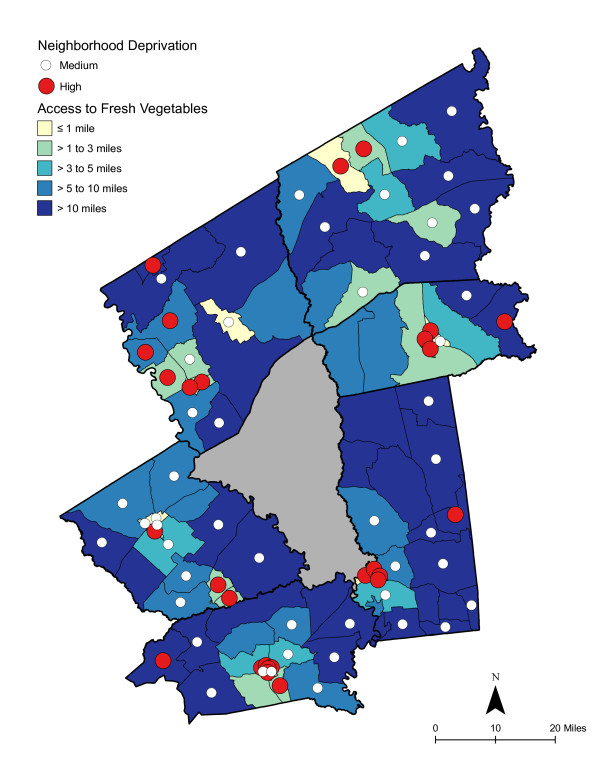
**Area-level Deprivation and Access to Fresh Vegetables**.

**Figure 5:**
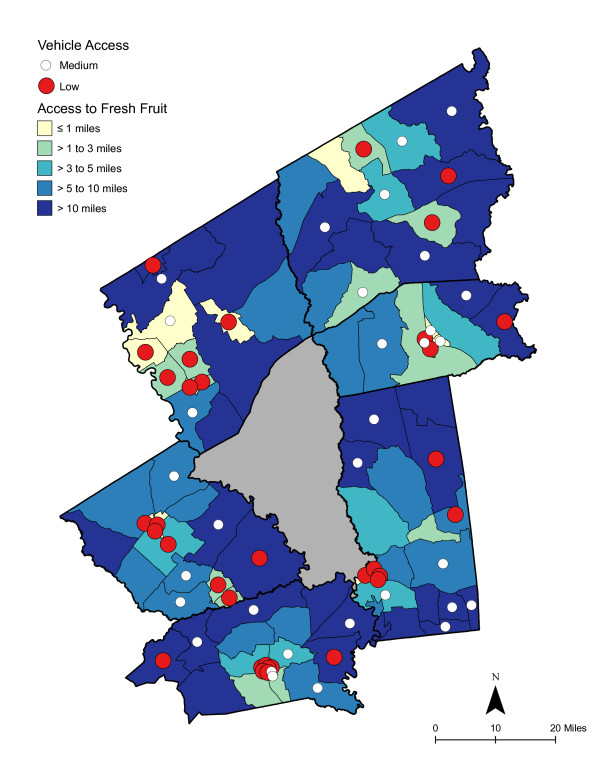
**Area-level Vehicle Ownership and Access to Fresh Fruit**.

**Figure 6:**
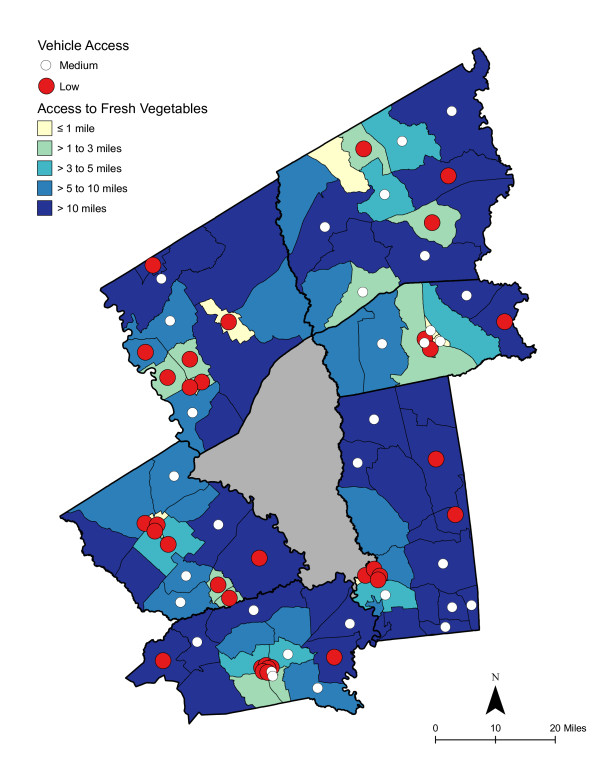
**Area-level Vehicle Ownership and Access to Fresh Vegetables**.

**Figure 7:**
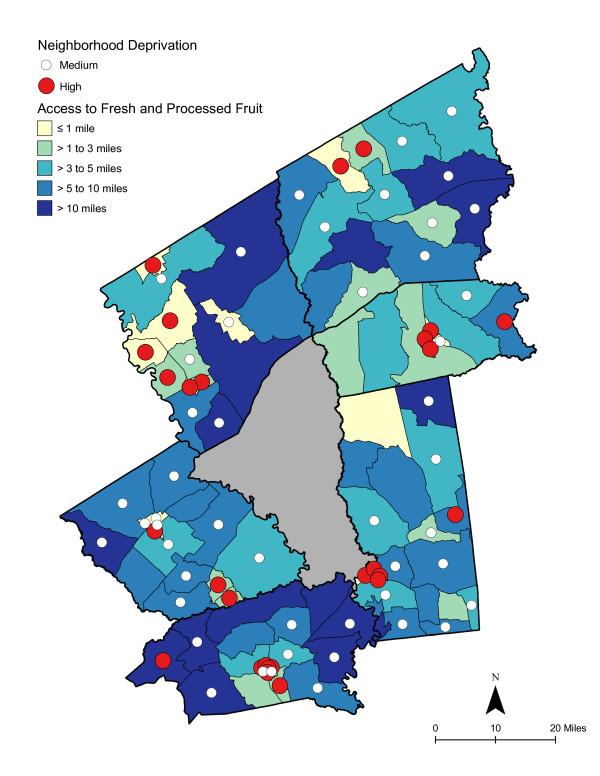
**Area-level Deprivation and Access to Fresh and Processed Fruit**.

**Figure 8:**
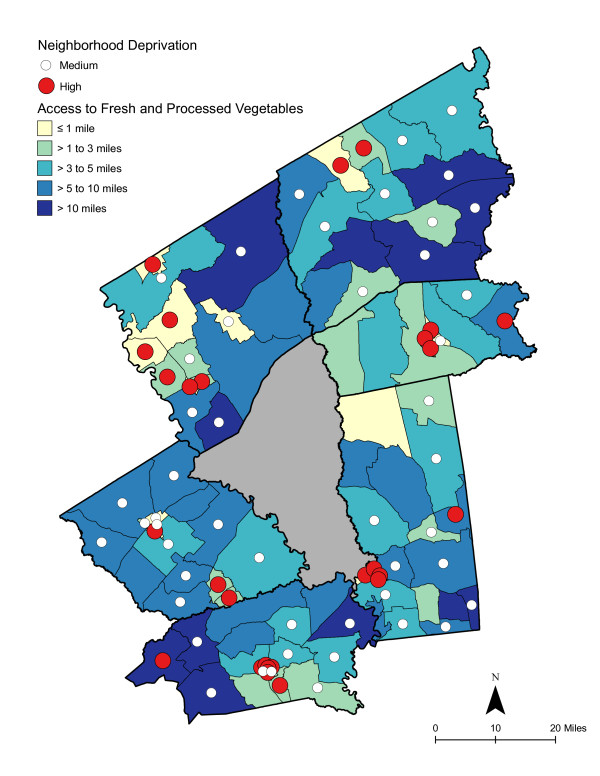
**Area-level Deprivation and Access to Fresh and Processed Vegetables**.

**Figure 9:**
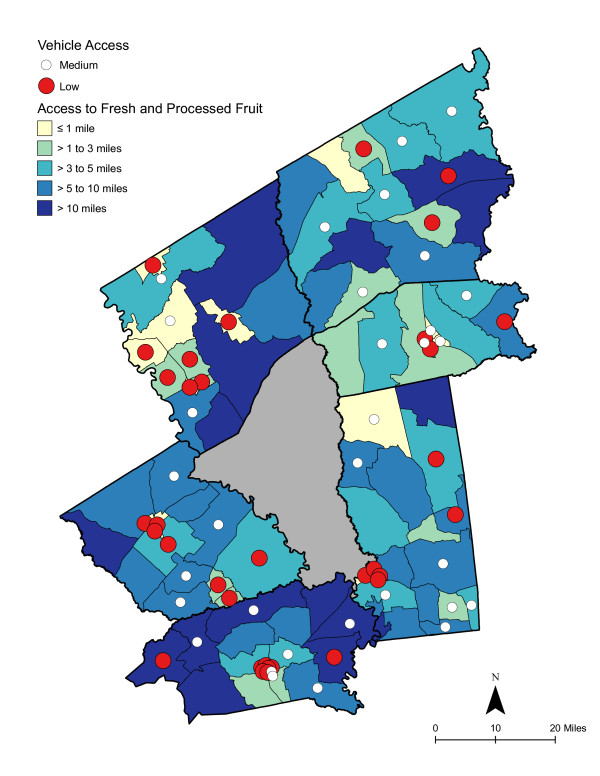
**Area-level Vehicle Ownership and Access to Fresh and Processed Fruit**.

**Figure 10:**
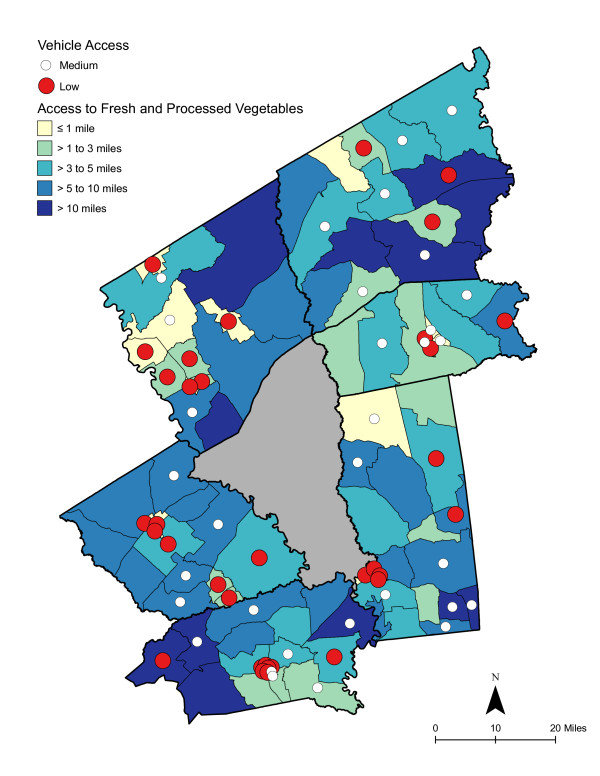
**Area-level Vehicle Ownership and Access to Fresh and Processed Vegetables**.

As shown in Table 6, access was best for high deprivation neighborhoods - in proximity and in the number of shopping opportunities. Overall, residents had to travel a shorter distance for a good variety of fresh fruits, fresh vegetables, fresh and processed fruits, or fresh and processed vegetables than to the nearest supermarket. The difference in distance to the nearest supermarket compared with the nearest food store with a good selection of fruits or vegetables remained significant for low and medium economically deprived neighborhoods; however, the differences were not statistically significant for high deprivation neighborhoods. Access, regardless of level of neighborhood deprivation, was better for a good variety of fresh and processed fruits or vegetables than for a good variety of fresh alone. Chloropleth maps (Figures 3 and 4) illustrate the spatial distribution of potential access to good varieties of fresh fruits and vegetables and neighborhoods with medium and high socioeconomic deprivation. The darkest color area indicates CBG that are greater than 10 miles one-way to the nearest food store with a good selection of fresh fruits (16.7% of medium and 11.1% of high deprivation areas) or vegetables (18.8% of medium and 14.8% of high deprivation areas). Table 7 and 8 show proximity and coverage of supermarkets, traditional food stores, and food stores with a good variety of fruits or vegetables by area-level vehicle ownership. Areas with lowest vehicle ownership had relatively better access to food stores and fruits or vegetables. Figures 5 and 6 indicate those areas with low (>9.5% of occupied households without a vehicle) or medium (4.6-9.5% without a vehicle) vehicle access. More than 20% of low vehicle access areas lacked access to fresh fruits and 23.5% to fresh vegetables within 10 miles. Chloropleth maps (see Figures 7, 8, 9, and 10) show access improved when fresh and processed fruits or vegetables were combined into overall fruits or vegetables. Still, there were areas where travel greater than 10 miles was necessary to access the nearest food store with a good variety of fresh or processed fruits or vegetables.

**Table 6:** Access to good availability of fresh and overall (fresh and processed) fruits and vegetables by neighborhood socioeconomic deprivation, using measures of proximity and coverage*

	All Deprivation (*n *= 101)	Low Deprivation (*n *= 26)	Medium Deprivation (*n *= 48)	High Deprivation (*n *= 27)
**Proximity (in miles)**				
***Supermarket***	9.9 ± 8.5	11.4 ± 8.8	12.1 ± 8.0	4.7 ± 6.8
***Fruits***				
Fresh fruits	6.7 ± 5.7^§^	8.1 ± 5.2^¶^	8.0 ± 5.4^§^	2.9 ± 5.1^‡^
Overall fruits	4.7 ± 4.2^§^	5.6 ± 3.8^¶^	5.8 ± 4.0^§^	2.0 ± 4.0^‡^
***Vegetables***				
Fresh vegetables	7.4 ± 6.1^§^	8.6 ± 5.6^¶^	8.8 ± 5.7^§^	4.0 ± 6.1^‡^
Overall vegetables	4.5 ± 4.1^§^	5.4 ± 3.6^¶^	5.3 ± 3.9^§^	2.1 ± 4.0^‡^
**Coverage - 1 mile**				
***Fruits***				
Fresh fruits	0.47 ± 0.82	0.65 ± 1.16	0.50 ± 0.99	1.89 ± 1.12^‡^
Overall fruits	0.77 ± 1.3	0.96 ± 1.51	0.79 ± 1.29	2.81 ± 1.73^‡^
***Vegetables***				
Fresh vegetables	0.37 ± 0.64	0.61 ± 1.1	0.35 ± 0.76	1.59 ± 1.08^‡^
Overall vegetables	0.59 ± 0.98	0.85 ± 1.22	0.69 ± 1.07	2.30 ± 1.23^‡^
**Coverage - 3 miles**				
***Fruits***				
Fresh fruits	0.91 ± 1.2	0.65 ± 1.16	0.50 ± 0.99	1.89 ± 1.12^‡^
Overall fruits	1.4 ± 1.7	0.96 ± 1.51	0.79 ± 1.29	2.81 ± 1.73^‡^
***Vegetables***				
Fresh vegetables	0.91 ± 1.2	0.61 ± 1.1	0.35 ± 0.76	1.59 ± 1.08^‡^
Overall vegetables	1.2 ± 1.3	0.85 ± 1.22	0.69 ± 1.07	2.30 ± 1.23^‡^
**Coverage - 5 miles**				
***Fruits***				
Fresh fruits	1.2 ± 1.3	1.0 ± 1.36	0.87 ± 1.18	1.89 ± 1.12^†^
Overall fruits	1.9 ± 1.8	1.5 ± 1.82	1.52 ± 1.61	2.85 ± 1.72^†^
***Vegetables***				
Fresh vegetables	0.93 ± 1.1	0.88 ± 1.27	0.58 ± 0.92	1.59 ± 1.08^†^
Overall vegetables	1.6 ± 1.4	1.30 ± 1.40	1.39 ± 1.35	2.37 ± 1.24^†^
**Coverage - 10 miles**				
***Fruits***				
Fresh fruits	2.1 ± 1.2	2.08 ± 1.29	1.90 ± 1.22	2.52 ± 1.19
Overall fruits	3.4 ± 1.8	3.58 ± 1.63	3.14 ± 1.80	3.70 ± 1.75
***Vegetables***				
Fresh vegetables	1.5 ± 1.0	1.65 ± 1.16	1.31 ± 0.93	1.81 ± 0.96
Overall vegetables	3.3 ± 1.5	3.35 ± 1.41	3.04 ± 1.58	3.59 ± 1.52

* Network distance shown as mean ± standard deviation; median in parenthesis

Level of statistical significance for test for trend across ordered groups of neighborhood socioeconomic deprivation: ^†^*p *< 0.01 ^‡^*p *< 0.001

Different than distance to nearest supermarket ^§^*p *< 0.001 ^¶ ^*p *< 0.01

**Table 7:** Spatial accessibility to fruits and vegetables by area-level vehicle ownership, using measures of proximity*

	Low Vehicle Ownership (*n *= 35)	Medium Vehicle Ownership (*n *= 32)	High Vehicle Ownership (*n *= 34)
**SPATIAL ACCESSIBILITY**			
***Proximity, mi***			
**Food stores**			
Supermarket	6.4 ± 7.8^§ ^(1.4; 0.3-23.8)	12.4 ± 8.4 (11.6; 0.6-30.9)	11.1 ± 8.2 (10.5; 0.1-33.6)
Traditional food store	4.1 ± 6.2^¶ ^(1.1; 0.1-23.1)	6.4 ± 7.8 (7.4; 0.6-24.4)	6.4 ± 7.8 (9.1; 0.1-19.0)
**Fruits**			
Fresh fruits	4.0 ± 5.5^§ ^(1.1; 0.1-19.5)	8.9 ± 6.2 (9.3; 0.8-19.8)	7.3 ± 4.3 (8.0; 0.1-15.1)
Overall fruits	2.7 ± 4.2^¶ ^(0.9; 0.1-19.5)	4.8 ± 3.3 (4.4; 0.5-12.4)	6.7 ± 4.1 (6.9; 0.1-14.6)
**Vegetables**			
Fresh vegetables	4.8 ± 6.7^§ ^(1.2; 0.4-23.1)	9.7 ± 6.5 (9.5; 0.8-23.5)	8.1 ± 4.5 (9.3; 0.1-16.3)
Overall vegetables	2.5 ± 3.9^¶ ^(1.0; 0.1-19.5)	5.3 ± 3.8 (4.5; 0.5-12.9)	5.7 ± 3.8 (5.6; 0.1-14.6)

Area-level (CBG) vehicle ownership (% owner-occupied households): Low = < 90.5%; medium = 90.5-95.4%; high = >95.4%. * Values calculated for each of the CBG (census block group) in the study area (*n *= 101). Proximity determined by the network distance from each CBG population-weighted centroid to the nearest food store. Distance (proximity) and percentages (mean ± standard deviation, median, and range) by category of vehicle ownership.

Level of statistical significance for test for trend across ordered groups of area = -level vehicle ownership: ^‡^*p *< 0.05 ^§^*p *< 0.01 ^¶^*p *< 0.001

**Table 8:** Spatial accessibility to fruits and vegetables by area-level vehicle ownership, using measures of coverage*

		Low Vehicle Ownership (*n *= 35)	Medium Vehicle Ownership (*n *= 32)	High Vehicle Ownership (*n *= 34)
**SPATIAL ACCESSIBILITY**				
***Coverage - 3 mi***				
**Food stores**				
	Supermarket	1.2 ± 1.1^¶ ^(1; 0-3)	0.4 ± 0.9 (0; 0-3)	0.5 ± 0.9 (0; 0-3)
	Traditional	1.6 ± 1.1^¶ ^(2; 0-2)	0.5 ± 0.9 (0; 0-3)	0.6 ± 1.0 (0; 0-3)
**Fruits**				
	Fresh fruits	1.7 ± 1.2^¶ ^(2; 0-3)	0.4 ± 0.9 (0; 0-3)	0.6 ± 1.1 (0; 0-3)
	Overall fruits	2.6 ± 1.8^§ ^(3; 0-6)	0.8 ± 1.1 (0; 0-3)	0.8 ± 1.4 (0; 0-6)
**Vegetables**				
	Fresh vegetable	1.3 ± 1.1^§ ^(1; 0-3)	0.4 ± 0.9 (0; 0-3)	0.5 ± 0.9 (0; 0-3)
	Overall vegetables	2.0 ± 1.4^¶ ^(2.5; 0-4)	0.7 ± 1.0 (0; 0-3)	0.8 ± 1.2 (0; 0-4)
***Coverage - 5 mi***				
**Food stores**				
	Supermarket	1.2 ± 1.1^‡ ^(1; 0-3)	0.6 ± 1.0 (0; 0-3)	0.7 ± 1.1 (0; 0-3)
	Traditional	1.7 ± 1.1^¶ ^(2; 0-3)	0.9 ± 1.0 (1; 0-3)	0.9 ± 1.2 (0; 0-3)
**Fruits**				
	Fresh fruits	1.8 ± 1.2^§ ^(2; 0-3)	0.8 ± 1.1 (0; 0-3)	0.9 ± 1.3 (0; 0-3)
	Overall fruits	2.7 ± 1.7^§ ^(3; 0-6)	1.5 ± 1.4 (1; 0-6)	1.3 ± 1.9 (0; 0-6)
**Vegetables**				
	Fresh vegetables	1.4 ± 1.1^‡ ^(1; 0-3)	0.7 ± 1.1 (0; 0-3)	0.7 ± 1.1 ()0; 0-3)
	Overall vegetables	2.3 ± 1.4^¶ ^(3; 0-4)	1.3 ± 1.1 (1; 0-4)	1.3 ± 1.4 (1; 0-4)
***Coverage - 10 mi***				
**Food stores**				
	Supermarket	1.5 ± 1.0 (2; 0-3)	1.2 ± 1.0 (1; 0-3)	1.5 ± 1.1 (1; 0-4)
	Traditional	2.3 ± 1.0 (3; 0-4)	1.7 ± 1.0 (2; 0-4)	2.2 ± 1.3 (2; 0-5)
**Fruits**				
	Fresh fruits	2.3 ± 1.3 (3; 0-4)	1.7 ± 1.1 (2; 0-3)	2.3 ± 1.3 (3; 0-4_
	Overall fruits	3.6 ± 1.8 (3; 0-7)	3 ± 1.5 (3; 1-7)	3.6 ± 1.9 (3; 0-8)
**Vegetables**				
	Fresh vegetables	1.6 ± 1.0 (2; 0-3)	1.3 ± 1.0 (1; 0-3)	1.6 ± 1.1 (2; 0-4)
	Overall vegetables	3.4 ± 1.6 (4; 0-6)	2.8 ± 1.3 (3; 1-5)	3.4 ± 1.5 (3; 0-6)

Area-level (CBG) vehicle ownership (% owner-occupied households): Low = <90.5%; medium = 90.5-95.4%; high = >95.4%. * Values calculated for each of the CBG (census block group) in the study area (*n *= 101). Coverage is determined by the number of food stores within a specific network-based distance. Numbers (coverage) and percentages (mean ± standard deviation, median, and range) by category of vehicle ownership

Level of statistical significance for test for trend across ordered groups of area = -level vehicle ownership: ^‡^*p *< 0.05 ^§^*p *< 0.01 ^¶^*p *< 0.001

### Multivariate models for access

Multivariate linear regression models were used to examine the relationship between neighborhood socioeconomic deprivation or vehicle ownership and access to a good variety of fruits or vegetables, controlling for population density. Table 9 shows that residents in high deprivation or low vehicle ownership areas, compared with low deprivation or high vehicle ownership areas, had to travel a significantly shorter distance to the nearest food store for fruits or vegetables. Population density was significant; the greater the population density, the better the access to the nearest fruits or vegetable opportunity. A similar relationship for 3-mile coverage is shown in Table 10 and for 5-mile coverage in Table 11. High deprivation or low vehicle ownership areas were associated with a greater number of shopping opportunities for fruits or vegetables. As with the prior analysis, increasing population density was similarly associated with greater coverage.

**Table 9:** Association between proximity to a good selection of fruits and vegetables and area deprivation or vehicle ownership, using multivariate linear regression model

Model 1	Access as network distance to the nearest			
	
	Fresh fruits	Overall fruits	Fresh vegetables	Overall vegetables
	
Deprivation	b (SE)	b (SE)	b (SE)	b (SE)
High	-4.47 (0.134)^‡^	-3.09 (1.02)^†^	-3.82 (1.44)^†^	-2.91 (1.0)^†^
Medium	-0.86 (1.19)	-0.33 (0.91)	-0.75(1.28)	-0.55 (0.89)

R^2^	0.303	0.264	0.291	0.243
*P*	<0.001	<0.001	<0.001	<0.001

				
	
**Model 2**	**Access as network distance to the nearest**			
	
	**Fresh fruits**	**Overall fruits**	**Fresh vegetables**	**Overall vegetables**
	
**Vehicle ownership**	**b (SE)**	**b (SE)**	**b (SE)**	**b (SE)**

Low	-2.26 (1.21)	-3.21 (0.90)^‡^	-1.93 (1.33)	-2.56 (0.89)^†^
Medium	1.23 (1.21)	-2.07 (0.90)^‡^	1.29(1.32)	-0.66 (0.89)

R^2^	0.269	0.276	0.268	0.233
*P*	<0.001	<0.001	<0.001	<0.001

NOTE: In models 1 and 2, the four equations were simultaneously estimated, controlling for population density. In model 1, deprivation entered as categorical variable; low deprivation is referent group. In model 2, vehicle ownership entered as categorical variable; high vehicle ownership is referent group. In both models, population density entered as continuous. Results are reported as multivariate-adjusted b (SE). Statistically significant variables are indicated as: *<0.05 ^†^<0.01 ^‡^<0.001

**Table 10:** Association between 3-mile coverage of a good selection of fruits and vegetables and area deprivation or vehicle ownership, using multivariate linear regression model

Model 1	Access as number of shopping opportunities within 3 network 3 miles			
	
	Fresh fruits	Overall fruits	Fresh vegetables	Overall vegetables
	
Deprivation	b (SE)	b (SE)	b (SE)	b (SE)
High	0.98 (0.21)^‡^	1.58 (0.34)^‡^	0.75 (0.18)^‡^	1.21 (0.25)^‡^
Medium	0.11 (0.18)	0.12 (0.30)	-0.02(0.16)	0.10 (0.22)

R^2^	0.633	0.497	0.660	0.562
*P*	<0.001	<0.001	<0.001	<0.001

				
	
**Model 2**	**Access as number of shopping opportunities within 3 network 3 miles**			
	
	**Fresh fruits**	**Overall fruits**	**Fresh vegetables**	**Overall vegetables**
	
**Vehicle ownership**	**b (SE)**	**b (SE)**	**b (SE)**	**b (SE)**

Low	0.69 (0.19)^‡^	1.37 (0.31)^‡^	0.50 (0.17)^†^	0.88 (0.23)^‡^
Medium	-0.05 (0.19)	0.13 (0.31)	0.02(0.17)	-0.01 (0.23)

R^2^	0.598	0.475	0.610	0.516
*P*	<0.001	<0.001	<0.001	<0.001

NOTE: In models 1 and 2, the four equations were simultaneously estimated, controlling for population density. In model 1, deprivation entered as categorical variable; low deprivation is referent group. In model 2, vehicle ownership entered as categorical variable; high vehicle ownership is referent group. In both models, population density entered as continuous. Results are reported as multivariate-adjusted b (SE). Statistically significant variables are indicated as: *<0.05 ^†^<0.01 ^‡^<0.001

**Table 11:** Association between 5-mile coverage of a good selection of fruits and vegetables and area deprivation or vehicle ownership, using multivariate linear regression model

Model 1	Access as number of shopping opportunities within 3 network 3 miles			
	
	Fresh fruits	Overall fruits	Fresh vegetables	Overall vegetables
	
Deprivation	b (SE)	b (SE)	b (SE)	b (SE)
High	0.98 (0.21)^‡^	1.58 (0.34)^‡^	0.75 (0.18)^‡^	1.21 (0.25)^‡^
Medium	0.11 (0.18)	0.12 (0.30)	-0.02(0.16)	0.10 (0.22)

R^2^	0.633	0.497	0.660	0.562
*P*	<0.001	<0.001	<0.001	<0.001

**Model 2**	**Access as number of shopping opportunities within 5 miles**			
	
	**Fresh fruits**	**Overall fruits**	**Fresh vegetables**	**Overall vegetables**
	
**Vehicle ownership**	**b (SE)**	**b (SE)**	**b (SE)**	**b (SE)**

Low	0.47 (0.25)	1.05 (0.38)^†^	0.32 (0.21)	0.76 (0.29)^†^
Medium	-0.03 (0.24)	0.28 (0.38)	0.07(0.21)	0.11 (0.29)

R^2^	0.402	0.267	0.438	0.314
*P*	<0.001	<0.001	<0.001	<0.001

NOTE: In models 1 and 2, the four equations were simultaneously estimated, controlling for population density. In model 1, deprivation entered as categorical variable; low deprivation is referent group. In model 2, vehicle ownership entered as categorical variable; high vehicle ownership is referent group. In both models, population density entered as continuous. Results are reported as multivariate-adjusted b (SE). Statistically significant variables are indicated as: *<0.05 ^†^<0.01 ^‡^<0.001

## Discussion

Findings from this study extend our understanding of potential spatial access from rural neighborhoods, not just to supermarkets, but to all food stores that market fruits and vegetables. We examined two dimensions of access: 1) proximity or distance to the nearest food store that offers a good variety of fruits or vegetables, and 2) coverage or the number of shopping opportunities for fruits or vegetables within a specified distance of the neighborhood. This is apparently the first study, to our knowledge, that uses ground-based data on the availability of fresh and processed (canned, frozen, and 100% juice) fruits and vegetables from traditional, convenience, and non-traditional food stores to examine access and availability of fruits and vegetables and the relationship between area inequalities (area-level socioeconomic deprivation or vehicle ownership) and access to fruits and vegetables, especially in a large rural area. Our analyses not only revealed that rural residents had relatively better access, in terms of closer distance and greater number of shopping opportunities, to a good selection of fruits or vegetables than to the nearest supermarket, but that access to fruits and vegetables was generally better for residents of high deprivation or low vehicle ownership neighborhoods. Several of the findings warrant further mention.

### Availability of fruits and vegetables

Fresh fruits and vegetables were available in all traditional food stores (supercenters, supermarkets, and grocery stores) and in none of the non-traditional food stores (dollar stores, mass merchandisers, and pharmacy); however, 6% (*n *= 9) of 140 convenience stores marketed both fresh fruits and vegetables and 22% marketed either fresh fruits or vegetables. The inclusion of healthier canned fruits expanded the picture of availability to include all non-traditional food stores and 48% of convenience stores. All non-traditional food stores and 90% of convenience stores marketed canned vegetables. The variety of fruits or vegetables was greater at supermarkets compared with grocery stores. Among non-traditional food stores, the largest variety was found at dollar stores, which was greater than that found at convenience stores. These results highlight the limitations of prior methods for examining fruit and vegetable availability which may misrepresent actual availability in a number of ways [17]. For example, prior work focused on supermarkets as sole source for fruits and vegetables and omitted non-traditional food stores, such as dollar stores or mass merchandisers, which are dramatically growing in numbers and increasing the opportunities for food and beverage shopping [65,90]. Over the past 10 years, dollar stores have increased the variety of lower-price shopping and food options to consumers [91-93]. According to the Nielsen Company, dollar stores are also attracting high and middle income shoppers, in addition to their primary customer - low-income shoppers [90]. In addition to the dynamics of the retail food environment, the impact of the economic downturn and increased vehicle costs may alter food shopping patterns. Prior examinations of fruit and vegetable access failed to include the presence of canned or frozen forms even though dietary recommendations specify fresh, canned, frozen, or 100% juice [70]. Furthermore, the variety (i.e., the number of different types of fruits or vegetables) is often not considered as part of availability, which results in a lack of differentiation between a location having one type of fruit compared with multiple types of fruits.

### Spatial access

Spatial access to a good variety of fresh or processed fruits or vegetables, using proximity (distance to the nearest food store) and coverage (number of shopping opportunities), was better for rural neighborhoods than access to the nearest supermarket. On average, rural neighborhoods (CBG) were 9.9 miles to the nearest supermarket, 7.0 miles to the nearest traditional food store (supercenter, supermarket, or grocery store), 6.7 miles and 7.4 miles to the nearest food store with a good variety of fresh fruits and vegetables, respectively, and 4.7 miles and 4.5 miles to a good variety of fresh and processed fruits or vegetables. As demonstrated in this study, the distance varied greatly depending whether access was to the nearest supermarket, the nearest food store regardless of type with a good variety fresh fruits or vegetables, or the nearest food store with a good variety of fresh and processed fruits or vegetables. Access to an available supply of fruits or vegetables provides a more realistic picture of rural access than distance alone to a supermarket or traditional food store, or store density for limited types of fresh fruits and vegetable [36,57]. Interestingly, the differences in distance remained significant for neighborhoods classified as having low or medium socioeconomic deprivation, but not for high deprivation, where proximity and coverage was generally best. High deprivation neighborhoods were located at a distance that would require access and resources for a car for transportation and where the largest percentage of occupied housing does not have an available vehicle. For neighborhoods with lower vehicle ownership, the median distance to a food store or selection of fruits or vegetables was beyond walking distance.

### Food deserts

Food deserts have been described as areas, particularly lower income neighborhoods, with limited access to affordable and nutritious food [17]. In small-town and rural areas, food deserts have been defined as areas more than 10 miles from a supermarket [55,94]. The 10 mile threshold is considered "somewhat arbitrary, considering that without a car, any distance of more than a mile or so could be considered unacceptably far" [17]. As shown in the choloropleth maps, there were several medium and high deprivation neighborhoods that could be described as food deserts, where residents lacked access to supermarkets or to fresh or processed fruits or vegetables [95]. Maps also illustrated vehicle ownership areas where food stores or supplies of fruits or vegetables were not accessible.

### Neighborhood Inequalities and Access

Finally, the findings from multivariate regression models confirmed that the most deprived or lowest vehicle ownership rural neighborhoods were not the most food-disadvantaged neighborhoods. Compared with low deprivation or high vehicle ownership neighborhoods, high deprivation or low vehicle ownership neighborhoods had better spatial access to a good variety of fruits and vegetables, both in the distance to the nearest source for fruits or vegetables and in the number of shopping opportunities, after controlling for the influence of neighborhood population density. Population density was included in the models, based on prior work in rural areas that found that food stores were located in closer proximity to neighborhoods or communities with greater population density [36,96].

It has been suggested that poorer quality neighborhoods amplify individual disadvantages through poorer access to healthy foods [21]. Key findings from this study add to the discussion and understanding of the influence of neighborhood inequalities on physical access to healthy foods. Access to food stores (primarily large supermarkets) has been studied in the U.S., U.K., Europe, Canada, Australia, and New Zealand, and with mixed results. In some studies, poor or minority areas provided little or no access to supermarkets [25,38,39,48,53,97-102]; other studies found little or no difference between deprived and affluent areas in access to supermarkets [37,59,71,78,103,104], or better access from deprived neighborhoods [36,60,61,104,105]. Importantly, we followed the recommendation of Macdonald and colleagues [61] that the nutritional value of the foods available in food stores should be considered and not just the proximity or density of retail food stores. In fact, the U.S. Department of Agriculture's food guidance system in its dietary recommendations for fruits and vegetables identify canned, frozen, and 100% juice in addition to fresh as a way to help people achieve the recommended variety and amount of fruits and vegetables [70]. Supporting this is the research that confirmed nutrient benefits of canned and frozen fruits and vegetables [69,71-77]. Our results show that, in this large rural area, the more disadvantaged neighborhoods had relatively better potential access to fresh fruits and vegetables and to combined fresh and processed fruits and vegetables than less disadvantaged neighborhoods. This suggests different processes operating in rural areas compared with large urban areas in U.S., many of which are socially and racially segregated.

Physical access to food stores has been shown to be a major problem for people in deprived communities; those without cars, older residents, people on low incomes, and residents of rural areas [36,47,48,53-57]. There may be challenges, even for those with relatively close potential access to healthy foods, due to the availability of a vehicle, lack of public transportation, limited financial resources (type, amount, timing, and competing demands), problems with the home environment (food storage, meal preparation area, and refrigeration), and the constraints of household size, and employment including location and work schedule. For example, limited household refrigeration may require a consumer to make frequent, costly trips for perishable food items; or purchase more expensive or less healthy food items from a retail store closer to home [14,30,31].

### Strengths

There are several major methodological strengths to this study. First, this study relied on the identification of all traditional, convenience, and non-traditional food stores through ground truthing, a methodology that we have previously shown to be more accurate in small-town and rural areas than secondary or publicly acquired lists [34,36]. Second, we included multiple store types, such as convenience stores, dollar stores, and mass merchandisers, which reflect a more realistic picture of potential retail food opportunities. Finally, availability and variety of fruits and vegetables were determined through a comprehensive on-site observational survey that included fresh, canned, frozen, and 100% juice forms of fruits and vegetables. This is in response to the criticism of others who posit that it is not enough to determine location of food stores in relation to neighborhoods without also considering the quality or healthiness of the food that is available [17,21,61].

### Limitations

Data allow us to examine potential spatial access, but do not capture purchase behavioral characteristics; that is, where and how frequently rural residents choose to shop for fruits and vegetables. An underlying assumption with most studies is that shopping trips originate from the residence; however, the starting point for food shopping may vary and depend on time and location of work or other activities in multiple stops that include food shopping [17,106]. Although we did not identify famer's markets or fruit/vegetable stands during our ground truthing, there was no measure of community or individual gardens. Future work with rural families will allow us to understand the role of non-retail sources for fruits or vegetables, such as food sharing, home gardens, and canning or freezing. The cost of collecting ground-based, comprehensive in-store data makes it difficult to replicate this study in numerous settings. However, we believe the value from the breadth and depth of data justifies the resources. Another limitation is the use of area-level data from the 2000 U.S. Census. We acknowledge that there have been changes since 2000; most notably, increased proportion of minority residents, greater unemployment, and increased proportion of low-income residents. We plan to update our work when data from the 2010 U.S. Census is released. Still, this is the best available source of CBG-level data. Finally, we are limited in our ability to generalize beyond our rural region. However, the use of all store formats and forms of fruits and vegetables has relevance to an international audience. Future plans call for a similar examination in small-town and rural areas outside the U.S.

## Conclusion

Despite these limitations, this study furthers our knowledge about access and availability of fruits and vegetables in retail food stores in a large rural area. This paper responded to the methodological challenges that have been identified in measuring potential access to food stores in rural areas [34]. The measurement of the food environment recognized the emergence of new and changing store formats. Supermarkets and grocery stores are no longer the only shopping opportunities for fruits or vegetables. Restricting shopping opportunities to supermarkets would understate the access to fruits or vegetables. Access was described in distance to the nearest food opportunity and cumulative opportunities or variety of opportunities within a specific geographic area [37]. Data on availability of fresh or processed fruits or vegetables in the measurements provide robust meaning to the concept of potential access in this large rural area. There remains an unanswered question. Do we think there are separate rural and urban definitions for access and availability? We posit that availability must include the target foods, such as fruits or vegetables. This is not a rural or urban construct; however, access as a distance measure does have different meanings. Although rural residents are more accustomed to travel than urban counterparts [17], vehicle ownership and time and resource costs must be included in a discussion of access. Future linking of utilization or realized access with potential access will enhance our understanding of access, as will a better understanding of individual or household travel patterns [107].

Access to a good variety of healthy foods, such as fruits and vegetables, can play a pivotal role in the nutritional health of rural families. Knowing more about the level of access to shopping opportunities for healthy foods is essential for combining environmental approaches with traditional health interventions to make it easier for individuals to make healthier food choices [106]. This is highly relevant for an international audience. Small-town and rural settings are not small versions of urban areas. Access to an available source of healthy foods, such as fruits and vegetables requires the identification of policy and environmental strategies that adapt a food systems approach to rural settings and involve stakeholders that affect production and consumption. Supply-side interventions could include strategies to increase access to healthy foods through the development of alternative strategies for providing high quality foods. At the same time, efforts should expand the focus from supply and pricing to the ability of rural residents to access and afford. Future work calls for an examination of utilization of retail food stores, especially the degree to which families frequent and purchase food items from convenience and non-traditional food stores.

## Competing interests

The authors declare that they have no competing interests.

## Authors' contributions

JRS developed the original idea for the study. JRS worked on the development of the instrument and the protocol for collection of data. SAH conducted all geocoding and mapping. JRS wrote the first draft of the paper. JRS, SAH, and WRD read and approved the final manuscript.
